# Videolaryngoscopy versus direct laryngoscopy for endotracheal intubation of cardiac arrest patients in hospital: A systematic literature review

**DOI:** 10.1016/j.resplu.2022.100297

**Published:** 2022-09-05

**Authors:** Lauren Cox, Alexandra Tebbett

**Affiliations:** Warwick Medical School, Gibbet Hill Road, Coventry CV4 7HL, UK

**Keywords:** Airway management, Cardiac arrest, Endotracheal intubation, Videolaryngoscopy

## Abstract

**Aims:**

Airway management during cardiopulmonary resuscitation may involve endotracheal intubation complicated by associated difficulties. Videolaryngoscopy may help to ease these difficulties and increase success rates by removing the need to achieve a direct line of sight required by standard direct laryngoscopy. This literature review aims to establish if there is an overall benefit in using videolaryngoscopy over direct laryngoscopy when intubating patients during cardiac arrest in the non-theatre hospital environment.

**Methods:**

The review was registered on PROSPERO (record ID 329987). A systematic search was conducted of EMBASE, MEDLINE, CINAHL and Web of Science for literature comparing the use of videolaryngoscopy to direct laryngoscopy during intubation of cardiac arrest patients in hospital up until 4th May 2022. The Cochrane Central Register of Controlled Trials (CENTRAL) database was accessed, and reference lists of relevant systematic reviews were analysed for further papers. Forward and backward citation tracking was carried out of the shortlisted papers to hand-search for any further relevant studies. Papers were included in the review if they used adult patients, the patients were intubated during cardiac arrest in hospital and if the papers were in English language or had an accessible translation. Papers were excluded if patients were intubated not during cardiac arrest, the studies were based outside of a hospital setting or in the operating theatre, the patients were paediatric or if the study used a simulation or manikin. The Critical Appraisal Skills Programme checklists were used to assess risk of bias. Odds ratios, confidence intervals and probability values were used to synthesise results.

**Results:**

Six studies were identified that collectively analysed 4525 patients who were intubated during cardiac arrest in the non-theatre hospital environment; five studies were observational and one a randomised controlled trial. Most of the studies being observational in nature led to a significant bias in their methodology which is a limitation to this review. The studies all measured first pass success rate as the primary outcome. First pass success rate only improved with videolaryngoscopy compared to direct laryngoscopy when the intubator was a less experienced clinician. Videolaryngoscopy also reduced some endotracheal intubation related complications and improved glottic visualisation when compared to direct laryngoscopy.

**Conclusion:**

The limited data suggests that use of videolaryngoscopy improved first pass success rates compared to direct laryngoscopy when the clinician was less experienced.

## Introduction

### Background

The European Resuscitation Council recommends that a stepwise approach is adopted to airway management during cardiopulmonary resuscitation (CPR) until effective ventilation is achieved.^1^ In some cases this may involve the placement of an advanced airway by inserting an endotracheal tube. CPR has been shown to increase the difficulty of endotracheal intubation (ETI), likely due to the motion artefact caused by chest compressions.^2^ As ETI during cardiac arrest is not a rare occurrence, it is important to find any approaches that may help to ease this difficulty.

In direct laryngoscopy (DL), a direct line of sight is required for glottic visualization, often requiring manipulation of the patient. Videolaryngoscopy (VL) uses video camera technology to help facilitate ETI by enabling the operator and other clinicians to visualise the airway structures on a screen.^3^ This technology means that alignment of the oral, pharyngeal and tracheal axes, and direct visualisation of the glottis are not required.^4^ There are many types of VL, each with different specifications. Some devices are akin to the conventional direct laryngoscope with a similarly shaped blade (McGrath Mac, C-MAC), others have a more angulated blade (GlideScope) and some include a channel for guiding the tube towards the glottis (Airway Scope).^3^

There has been an increase in the use of VL during the covid-19 pandemic due to infection prevention measures; VL enables the operator to maintain a greater distance between themselves and the patient’s airway.^5^ With VLs now being commonplace across hospitals in the UK, it needs to be determined if there are any overall benefits to the patient using VL over DL during cardiac arrest.

Previous literature reviews have compared the use of DL and VL when intubating patients in theatre6., 7. and found that VL leads to a greater first pass success (FPS) rate and reduced intubation failures. Arulkumaran et al.^8^ found VL improved FPS when used in the intensive care unit, but not in patients intubated during cardiac arrest (although the number of patients in this subgroup were low). These literature reviews all showed greater success rates when the C-MAC laryngoscope was used. There has not yet been a literature review that solely examines the success rates when VL is used during a cardiac arrest in hospital.

One of the most important factors that influences outcomes of cardiac arrests is the quality of the chest compressions. Even a brief interruption to chest compressions can cause a significant drop in perfusion pressure, which in turn is associated with a poorer outcome.^9^ Interrupting chest compressions for airway management purposes happens,10., 11. with pauses increasing if repeated attempts are needed to secure an airway.^12^ As using VL could help to improve visualisation of the glottis compared with DL,^13^ it may reduce both the time taken to intubate and the number of attempts needed, and thus reduce the risk of interruptions to chest compressions.

The aim of this literature review is to focus on intubations during adult cardiac arrest management in the non-theatre hospital environment, to see if there is an overall benefit in using VL over standard practice of DL with regards to ETI success, in particular FPS rates. It will be considered if there is any evidence for the superiority of one VL over another by assessing the brands used in each study, and if this had an impact on the success rates of VL compared to DL.

## Methods

### Search strategy

This systematic review was accepted for registration on PROSPERO on 16th May 2022 at https://www.crd.york.ac.uk/prospero/display_record.php?RecordID=329987. Though PROSPERO registration was made after the initial search, no changes were made to the protocol (Appendix 1).

An initial systematic search was conducted of EMBASE, MEDLINE, CINAHL and Web of Science for relevant literature up until 15th November 2021; this search was then updated on 4th May 2022. The search strategy was reviewed by an expert librarian and is shown in Appendix 2. The Cochrane Central Register of Controlled Trials (CENTRAL) database was used to search through reference lists of relevant systematic reviews on similar topics to ensure no papers were missed. Forward and backward citation tracking was carried out of the shortlisted papers to hand-search for any further relevant studies. The following search terms were used: videolaryngoscop*, GlideScope, Airtraq, X-Lite, Storz, McGrath, Pentax, C-MAC, direct laryngoscop*, laryngoscope, laryngoscopy, Macintosh, intubation, orotracheal, endotracheal, intratracheal, airway management, cardiac arrest, resuscitation, cardiopulmonary resuscitation, CPR, heart arrest and chest compressions. A search of ongoing trials was also conducted on 4th May 2022 using https://www.https://www.clinicaltrials.gov, but no relevant studies were found.

### Study selection

Two investigators independently screened the titles and abstracts of the references obtained from the literature search to find potentially relevant studies. Full text papers were then retrieved and assessed for eligibility by the two authors based upon the pre-determined inclusion and exclusion criteria. The following inclusion criteria was used: patients intubated during cardiac arrest in hospital, studies in English language or with an accessible translation and studies using adult patients. The following exclusion criteria was used: patients intubated not during cardiac arrest, studies that were based outside of a hospital setting, studies in the operating theatre, studies using paediatric patients and studies using simulation or manikins.

Once the final studies were selected, the Critical Appraisal Skills Programme (CASP) checklists^14^ were used to assess the quality and highlight any potential bias before extracting relevant data for analysis. These checklists consist of 11–12 questions that provide guidance on how to systematically appraise research papers, and they cover three main areas: validity, results, and clinical relevance. The results are shown in Appendix 3.

### Data collection and analysis

To ensure that the same information was obtained from each paper, a form was created to aid in data extraction (Table 1). The following data was collected: first author, publication year, country of study, number of centres in the study, area of hospital studied, timeframe of the study, data collection method, level of experience of the clinician intubating, type of VL used, type of DL used, number of patients assigned to each group, primary outcome and any secondary outcomes. Any funding declared was also noted for transparency. Data extraction was completed by the first author and reviewed by the second author to reduce the risk of mistakes or omissions.

**Table 1:** Study characteristics.

Author (publication year)	Country	Area of hospital	Duration	Data collection method	Level of doctor intubating	Type of VL^1^	Type of DL^2^	No. VL pts	No. DL pts	No. of centres per study	Funding
Min et al. (2019)^15^	Korea	Emergency department	April 2014 - July 2018	Retrospective study (cohort)	Junior resident, senior resident, attending physician	C-MAC	Macintosh	263	310	1	None
Kim et al. (2016)^19^	Korea	Emergency department	June 2011 - May 2013	Prospective randomised controlled study	Experienced intubator - >50 successful ETIs^3^	GlideScope	Not stated	71	69	1	None declared
Okamoto et al. (2019)^17^	Japan	Emergency department	February 2012 - November 2017	Prospective observational study	Transitional year resident, emergency medicine resident, attending emergency physician, other specialty	C-MAC, McGrath, AirwayScope, GlideScope	Not stated	613	2747	15	Support by grant of the St. Luke's Life Science Institute
Lee et al. (2015)^16^	Korea	Wards, study rooms, outpatient department., car park	January 2011 - December 2013	Retrospective study	Experienced – licensed medical or surgical specialist in critical care > 1 year, inexperienced – did not meet the above criteria	GlideScope, Airway Scope	Not stated	121	108	1	None
Khandelwal et al. (2014)^2^	United States of America	Out of operating room	January 2008 - December 2012	Retrospective study	Anaesthetist or ED doctor/trainee	GlideScope	Not stated	7	133	1	None declared
Park et al. (2015)^18^	Korea	Emergency department	May 2011 - April 2013	Prospective comparative study	First year residents	GlideScope	Not stated	49	34	1	None declared

1 Videolaryngoscope.

2 Direct laryngoscope.

3 Endotracheal Intubations.

To enable comparison between the results the reported FPS rates using both VL and DL from each study were used to calculate odds ratios, confidence intervals and probability values, using MedCalc® (Version 20.113, Belgium).^15^ A meta-analysis of the combined results was planned, if possible.

## Results

### Literature search

**Fig. 1:**
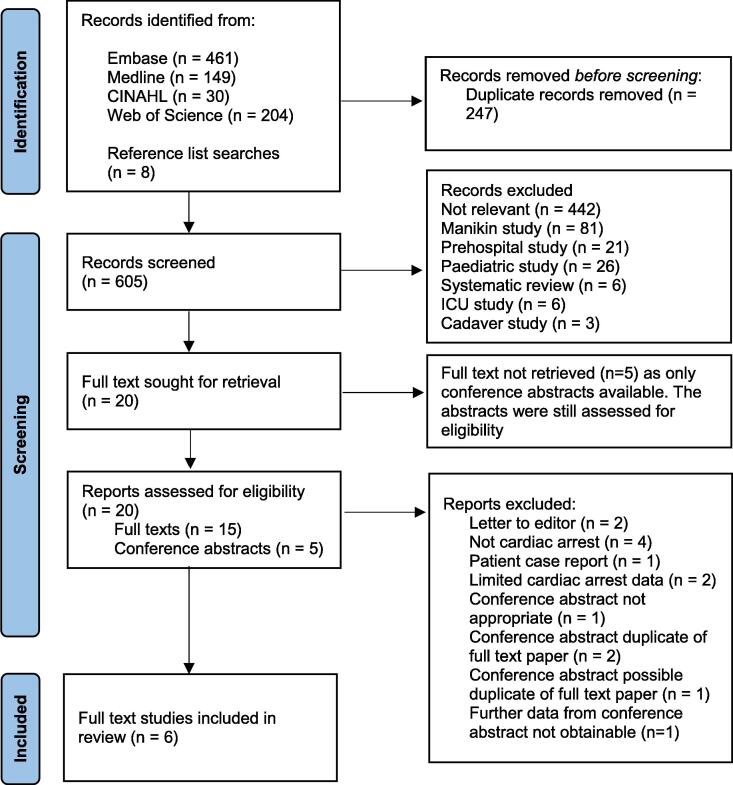
Preferred Reporting Items for Systematic Reviews and Meta-Analyses (PRISMA) diagram.

The search strategy produced a total of 852 potential papers from database searches (n = 844) and hand-searching reference lists (n = 8). Duplicates were removed (n = 247) and the remaining 605 papers were screened for eligibility. 585 papers were excluded for reasons detailed in the PRISMA checklist, Fig. 1, resulting in 20 papers sought for full text review. The further exclusion of 14 papers resulted in six included studies.2., 16., 17., 18., 19., 20. Journal publishers were contacted to acquire further information regarding eligible conference abstracts, but the data was not obtainable and the abstracts were removed.

### Study characteristics

A total of six studies were analysed, which together included 4525 patients that were intubated during cardiac arrest in a hospital setting. Details of these studies are shown in Table 1. Three were retrospective observational studies,2., 16., 17. two were prospective observational studies18., 19. and one was a prospective randomised controlled study.^20^

Most of the studies took place in the emergency department, with two studies differing from this. Khandelwal et al.^2^ enrolled any patient in cardiac arrest outside of the operating room, and Lee et al.^17^ included patients in cardiac arrest on a general ward, diagnostic study room, haemodialysis room, out-patient department, car park or hospital lobby. It could be argued that some of these locations do not count as in-hospital, however the authors included them as on site in their study and recorded the information about the arrests on hospital medical records, so they were included in this review.

A variety of brands of VL were used throughout the six studies, with some studies using more than one brand.17., 18. The most common VL to be used was the GlideScope, followed by the C-MAC, Airway Scope and McGrath. The type of DL was only specified in one of the studies as being the Macintosh.^16^ From these limited studies, there was no evidence of superiority of one type of VL over another.

There was a large disparity between the intervention (VL) and control (DL) group sizes when compared as a whole. Across the six studies and 4525 patients, a total of 1124 (25 %) patients were intubated using VL whereas 3401 (75 %) patients were intubated using DL. Three of the studies maintained a proportionate split between the groups,16., 17., 20. and three had a very uneven split between intervention and control.

The experience of clinician that performed the intubation varied throughout the studies, with Park et al.^19^ focussing on novice physicians, Kim et al.^20^ using clinicians they classified as being experienced, and the remaining studies using clinicians with a variety of experiences. The way in which clinicians were classified as experienced also varied but was usually based on their level of training. Kim et al.^20^ stated that intubations were usually performed by emergency physicians and classified someone as experienced if they had completed more than 50 previous intubations.

### Quality of included studies

Due to the nature of this area of research observational studies are more commonplace and this style of research has the potential to impart bias.^21^ Four out of the six papers had a high risk of selection bias due to the absence of randomisation; it was down to the clinicians’ personal choice as to which type of laryngoscope they intubated with.2., 16., 17., 18. All studies were at risk of performance bias as it was not possible to blind the clinicians to the type of laryngoscope they were using.

## Primary and secondary outcomes

All the studies looked at FPS rate as their primary outcome. There were a variety of secondary outcomes including: glottic visualisation, number of intubation attempts, total time to intubate, intubation failure, complications associated with intubation, return of spontaneous circulation (ROSC) and mortality rates. These outcomes are displayed in Table 2.

**Table 2:** Primary and secondary outcomes, FPS rates and statistical significance of included studies as described by the original authors.

Author (Publication year)	Primary Outcome	Secondary outcomes	FPS rate with VL^1^ n/N (%)	FPS rate with DL^2^ n/N (%)	Reported p-value
Min et al. (2019)^15^	FPS^3^ rate	Glottic visualisation, multiple attempts rate, ETI^4^-related complications (oesophageal intubation, dental injury, ROSC^5^, 24hr mortality, survival-to-discharge)	207/263 (79 %)	224/310 (72 %)	0.075
Kim et al. (2016)^19^	Success rate of ETI	No. of successful ETI attempts, total time to complete ETI, complications (oesophageal intubations, tooth injuries, chest compression interruption, serious no-flow)	123/128 (96 %)	109/124 (88 %)	Not specified. Described as significant for experienced only
Okamoto et al. (2019)^17^	FPS rate	Glottic visualisation, oesophageal intubation	480/613 (78 %)	1913/2747 (70 %)	<0.001
Lee et al. (2015)^16^	FPS rate	Time to ETI, ROSC, 24hr mortality, 28d mortality	87/121 (72 %)	57/108 (53 %)	0.003
Khandelwal et al. (2014)^2^	Odds of encountering a difficult intubation, FPS rate	Oesophageal intubation	35/49* (71 %)	291/371* (78 %)	0.27
Park et al. (2015)^18^	FPS rate	Time to ETI, chest compression interruption, oesophageal intubation	45/49 (92 %)	19/34 (56 %)	<0.001

*Included non-cardiac arrest patients in their statistical analysis.

1 Videolaryngoscope.

2 Direct laryngoscope.

3 First Pass Success.

4 Endotracheal Intubation.

5 Return of spontaneous circulation.

### First pass success

Of the six studies, four found a significant improvement in FPS rate when patients were intubated by a clinician using VL over DL.16., 17., 18., 19. Lee et al.^17^ found a significant improvement in FPS rate amongst all included intubations, regardless of clinician experience. For the other three the improvement in FPS rate was only when the intubation was performed by a clinician who was classified as less experienced, such as a junior resident (Min et al.),^16^ transitional-year resident (Okamoto et al.)^18^ or first-year resident (Park et al.)^19^ Khandelwal et al.^2^ classified most of their clinicians as experts and concluded that there was no significant difference in FPS rate between VL and DL, but the proportional split of their study groups was very uneven with only 5 % (n = 7) of their patients intubated during cardiac arrest using VL. A breakdown of FPS rates and, where specified, the significance value (p-value) can be found in Table 2.

**Fig. 2:**
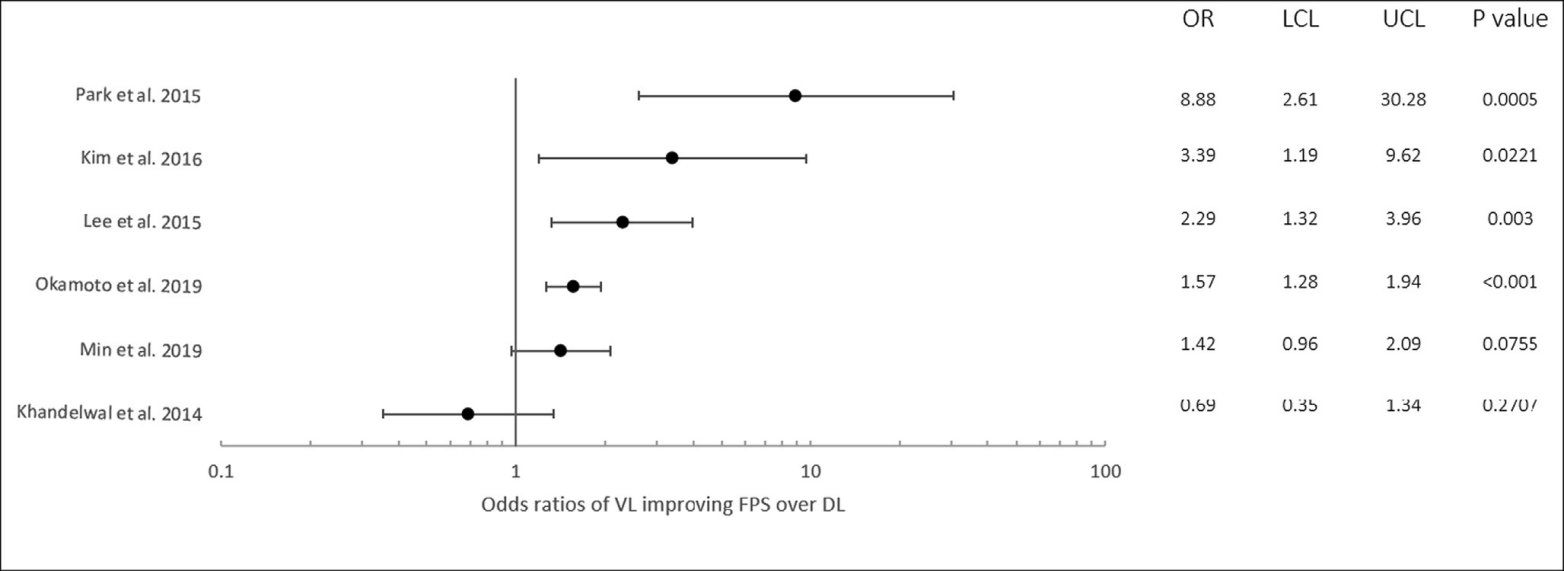
A Forrest Plot showing the calculated odds ratio and confidence intervals for the odds of videolayngoscopy (VL) over direct laryngoscopy (DL) in improving first pass success (FPS) intubation for the six studies.

As the studies utilised various statistical methods in measuring their effect size, new odds ratios and confidence intervals were calculated for comparison. These values are shown diagrammatically and numerically in Fig. 2, as the odds of VL improving FPS over DL.

A meta-analysis was originally considered but was not possible due to there being significant heterogeneity in the study definitions, methods, and outcomes. The six studies focused on clinicians with varied levels of experience, and had different methods of defining that experience. The type of intervention, the VL, varied. Three studies were retrospective and three were prospective, with the clinicians in the prospective studies assigned to use either VL or DL only, meaning it could be the clinicians being compared rather than the type of laryngoscope. Two studies utilised randomisation whereas the remaining four allowed for clinician choice in laryngoscope type. Whilst FPS was the primary outcome, definitions of FPS were not standardised and the way FPS was recorded varied.

### Glottic visualisation

In the two papers that investigated the effect that VL had on glottic visualisation, the Cormack-Lehane (C-L) grading system was used. C-L grading is a way to classify the view seen when performing laryngoscopy. It varies from grade 1 where most of the glottis is visible, to grade 4 where neither the glottis nor epiglottis is visible.^22^ Glottic visualisation was recorded by the intubating clinician when using either VL or DL. An improved glottic visualisation was classified as the operator obtaining a grade 1 view^18^ or either a grade 1 or 2 view^16^ when using VL. These results were then compared with the DL group as a whole and it was determined that VL improved glottic visualisation. This also correlated with an improved FPS rate when the operator was less experienced.16., 18.

### Complications

As well as assessing FPS, all the studies considered complications relating to ETI. Collectively it was found that the use of VL reduced the number of oesophageal intubations16., 18., 19. and reduced the time to intubate.^19^

Kim et al.^20^ and Park et al.^19^ discovered a reduced interruption to chest compressions when VL was used. They also found that DL was the only method of intubation that caused a serious no-flow event, classified as a pause of > 10 s in chest compressions, even when the operator was an experienced clinician. The use of either VL or DL had no significant effect on ROSC or mortality at 24 hours.16., 17.

## Discussion

### Key findings

A systematic review was carried out and retrieved six studies for analysis on whether the use of VL over DL provided any benefits when utilised for ETI during cardiac arrest in hospital. The main finding was that VL improved FPS rate when used by clinicians that had less experience intubating patients. It was also found that the use of VL improved glottic visualisation compared DL, though this may not necessarily improve intubation success. Once the view is obtained an endotracheal tube needs to be passed, which may remain difficult due patient position or chest compressions.^2^

ETI related complications were also evaluated. Most notably, the use of VL instead of DL showed a reduced interruption to chest compressions in the two studies that looked at this outcome. This could help to preserve the perfusion pressure and potentially improve cardiac arrest outcomes, however evidence of this was not shown as there was no effect on mortality outcomes when VL was used.

Most papers in this literature review were observational studies. The only RCT did not show any statistical significance for improved FPS rate when VL was used. The only significant finding was that of a reduced compression interruption when VL was used compared to DL.^20^ A variety of different types and brands of VLs were used throughout the studies and there was no specific VL that showed superiority over the others.

### Comparison to previous reviews

A previous systematic review by Arulkumaran et al.^8^ noted similar results when patients were intubated for emergency reasons outside the operating room. They discovered an advantage in using VL when used by novice clinicians, but not when used by those more experienced. There was a small number of patients that were intubated during cardiac arrest and VL did not improve FPS rates in this group.

A recent Cochrane review compared different types of VLs to DL and found that all designs of VLs were likely to improve FPS rates when used to intubate patients.^23^ However, most of the included studies were adults undergoing elective surgery, with only eight of the 222 studies including patients in cardiac arrest. Of these eight studies, three did not meet our inclusion criteria (they took place in the pre-hospital setting) and four would have done but the authors did not separate out the data for patients in cardiac arrest. The last study, Kim et al. (2016)^20^ was included in this literature review. No analysis was performed for patients in cardiac arrest so no conclusions about this subgroup can be made. This literature review builds upon the Cochrane review in this respect and includes five observational studies that the Cochrane review didn’t analyse.

### Strengths and limitations

The main strength of this systematic literature review is that it is the first of its kind to solely focus on the intubation of patients during cardiac arrest. It highlights areas of improvement for future research, including where standard definitions would be beneficial to help achieve homogeneity throughout the studies and improve the possibility of a future meta-analysis. This review shows benefits across multiple studies but not to a significant extent, possibly due to the small patient numbers in the intervention groups, and so highlights where further research could be focussed.

Two published conference abstracts24., 25. met the inclusion criteria for this review. However due to the limited information available in the abstracts they could not be formally assessed and so weren’t included. If the abstracts were included the overall results of this review would not have changed. Kim et al.^24^ found that VL did not improve FPS rates but did reduce the number of failed intubations, and Shirakura et al.^25^ found that VL did not improve FPS rates, although it was not stated if this was the case for all levels of operator experience. It is possible that Shirakura et al.^25^ evaluated the same population as one of the full text papers (Okamoto et al.),^18^ due to overlapping time periods and similar co-authors.

This review has limitations. Firstly, most of the studies included were observational in nature which presented significant bias when assessing the methodology used. There were few studies available in this area of research, and the ones that were included did not have many patients in the intervention (VL) group to allow for an accurate comparison. This results in an inability to offer recommendations for future practice at this stage.

The definition of an expert varied throughout the studies. It wasn’t clarified if the clinicians that were classified as experts had equivalent experience in both devices that they were using. They may be an expert in using traditional DL but may not have as much experience using VL due to it being a more modern technology. This could have caused a bias towards the DL in this specific group of clinicians.

Five of the six studies did not specify which type of DL was used in their control group, and so it cannot be completely certain what VL is being compared against. It could be assumed that the Macintosh was used due to it being the most commonplace DL.

Finally, when completing this type of research involving airway management, it is hard to account for those patients who may have difficult airways. There are also many other important factors to consider that contribute to a successful intubation, such as if the intubating clinician had someone assisting and how experienced they were, if adjuncts were available and were they utilised and was the correct equipment readily available in the areas where the intubations were performed. All these factors impact upon the ease of airway management and were often not accounted for in the studies.

### Implications for practice and future research

In the UK it is recommended that only experts with a high ETI success rate should intubate a patient during cardiac arrest.^1^ Using either VL or DL to intubate during these circumstances requires a similar set of skills and experience. As the results of this systematic review show no benefit when VL is used over DL by experienced clinicians, there is insufficient evidence to make any recommendations to change current practice. There was also no evidence on overall outcome measures such as morbidity and mortality. The current adult advanced life support guidelines state that a supraglottic airway can be used as an alternative to ETI,^1^ but this technique has not been looked at in this literature review.

Future research is required for a more thorough assessment of the use of VL in ETIs during cardiac arrests in hospital. There is a need for more RCTs in this area of research to minimise the risk of bias that is currently present, but this comes with its own complications. A greater number of patients are needed in the VL group so that a more accurate comparison can be obtained. A universal definition of experience is required so that the studies can be analysed together to strengthen the results and minimise bias.

## Conclusion

This literature review identified six papers that evaluated the use of VL compared to DL during intubation of cardiac arrest patients in hospital. There was significant heterogeneity of the included papers meaning that a formal meta-analysis was not possible, and so the lack of pooled outcomes, effects and estimates reduced the certainty of the evidence and the conclusions that can be made. For this reason, it was difficult to achieve a definitive answer to the main objective of this study, whether VL provides an overall benefit compared to the use of DL during cardiac arrest intubation. This literature review cannot make any recommendations to change current practice but highlights areas for further research and the need for standard definitions and clarity of practice to strengthen the available evidence.

## CRediT authorship contribution statement

**Lauren Cox:** Conceptualization, Methodology, Software, Data curation, Writing – original draft. **Alexandra Tebbett:** Conceptualization, Methodology, Supervision, Validation, Writing – review & editing.

## Declaration of Competing Interest

The authors declare that they have no known competing financial interests or personal relationships that could have appeared to influence the work reported in this paper.
