# Supplementary material for: Videolaryngoscopy versus direct laryngoscopy for endotracheal intubation of cardiac arrest patients in hospital: A systematic literature review
Source: Resusc Plus. 2022 Sep 5;11:100297. doi: 10.1016/j.resplu.2022.100297 (PMC9468586; doi:10.1016/j.resplu.2022.100297)
Supplement: Supplementary data 1 [file mmc1.docx]

**Videolaryngoscopy versus direct laryngoscopy for endotracheal intubation of cardiac arrest patients in hospital: a systematic review**

*Lauren Cox, Alex Tebbett*

**Citation**

Lauren Cox, Alex Tebbett. Videolaryngoscopy versus direct laryngoscopy for endotracheal intubation of cardiac arrest patients in hospital: a systematic review. PROSPERO 2022 CRD42022329987 Available from: <https://www.crd.york.ac.uk/prospero/display_record.php?ID=CRD42022329987>

**Review question**

Is there an overall benefit in using videolaryngoscopy over standard practice of direct laryngoscopy with regards to endotracheal intubation success during cardiac arrests in hospital?

**Searches**

EMBASE, MEDLINE, CINAHL, Web of Science. Original search 15/11/2021.

**Types of study to be included**

There are no restrictions to the study designs eligible for inclusion in this review.

**Condition or domain being studied**

The airway management of cardiac arrests

**Participants/population**

Inclusion: patients intubated during cardiac arrest in hospital, adult patients

Exclusion: patients intubated not during cardiac arrest, paediatric patients

**Intervention(s), exposure(s)**

Patients must be intubated using a videolaryngoscope during cardiac arrest, any type of videolaryngoscope was accepted.

**Comparator(s)/control**

The control is the use of direct laryngoscopy for intubation during cardiac arrest.

**Context**

Only studies that include patients intubated during cardiac arrest in hospital will be included. Intubation during anaesthesia in theatres will be excluded.

**Main outcome(s)**

This review will look at the use of videolaryngoscopy over direct laryngoscopy, to see if this technique improves first pass success rate of intubation during cardiac arrest.

**Additional outcome(s)**

This review will also look at if videolaryngoscopy reduces the number of intubation attempts, and if it is shown that there is a superiority of one videolaryngoscope over another.

**Data extraction (selection and coding)**

Two reviewers will be assessing the studies against the eligibility criteria and selecting studies for inclusion, the records will be individually screened using Rayyan.

The data to be extracted from each paper are as follows: Country of research, area of hospital, duration of study, data collection method, level of doctor intubating, type of videolaryngoscope, type of direct laryngoscope, number of patients intubated using videolaryngoscopy, number of patients intubated using direct laryngoscopy.

The data will be extracted by the first author and checked by the second author. The data will be recorded in an excel spreadsheet.

In the case of unreported data, study investigators will be attempted to be contacted for additional details.

**Risk of bias (quality) assessment**

When assessing for risk of bias, the methods of randomisation will be assessed, along with the blinding of clinicians to the intervention. It will be taken into account the type of study design that has been used and how this could impart bias on the research.

**Strategy for data synthesis**[1 change]

Due to the significant heterogeneousity in study designs and outcomes expected, a meta-analysis is unlikely to be suitable. Instead, a formal narrative synthesis will be performed, looking at the main outcome of first pass intubation success rates between direct laryngoscopy and videolaryngoscopy. Secondary outcomes such as number of intubation attempts, pause length in CPR and complications will also be analysed as the data allows. Patient outcome data, if available, will be commented on.

All studies that meet the inclusion/exclusion criteria will be included, and details of the authors, study aims, methods, comparisons and reported outcomes will be presented visually in tables. Any quantitative analysis performed by the original authors will be included, as will their statistical methods and conclusions. As mentioned, it is unlikely quantitative collective synthesis will be appropriate. The results will instead be discussed in the narrative collectively where able (for instance it is likely that we will be able to collectively analyse first past success rates across the majority of the included studies), and any emergent patterns in the results highlighted and possible conclusions discussed.

**Analysis of subgroups or subsets**

It will be assessed if the use of videolaryngoscopy as a whole improves the intubation success during cardiac arrest, but it will also be assessed if there are any differences in success rates when used by senior clinicians compared to junior clinicians.

**Contact details for further information**

Lauren Cox

laurencox@hotmail.co.uk

**Organisational affiliation of the review**

None

**Review team members and their organisational affiliations**

Miss Lauren Cox. Warwick Medical School
Dr Alex Tebbett.

**Type and method of review**

Intervention, Narrative synthesis, Systematic review

**Anticipated or actual start date**

15 November 2021

**Anticipated completion date**[2 changes]

02 July 2022

**Funding sources/sponsors**

None

**Conflicts of interest**

**Language**

English

**Country**

England

**Stage of review**

Review Ongoing

**Subject index terms status**

Subject indexing assigned by CRD

**Subject index terms**

Heart Arrest; Hospitals; Humans; Intubation, Intratracheal; Laryngoscopy

**Date of registration in PROSPERO**

16 May 2022

**Date of first submission**

03 May 2022

Appendix 1: The study protocol as registered on PROSPERO (<https://www.crd.york.ac.uk/prospero/display_record.php?ID=CRD42022329987>)

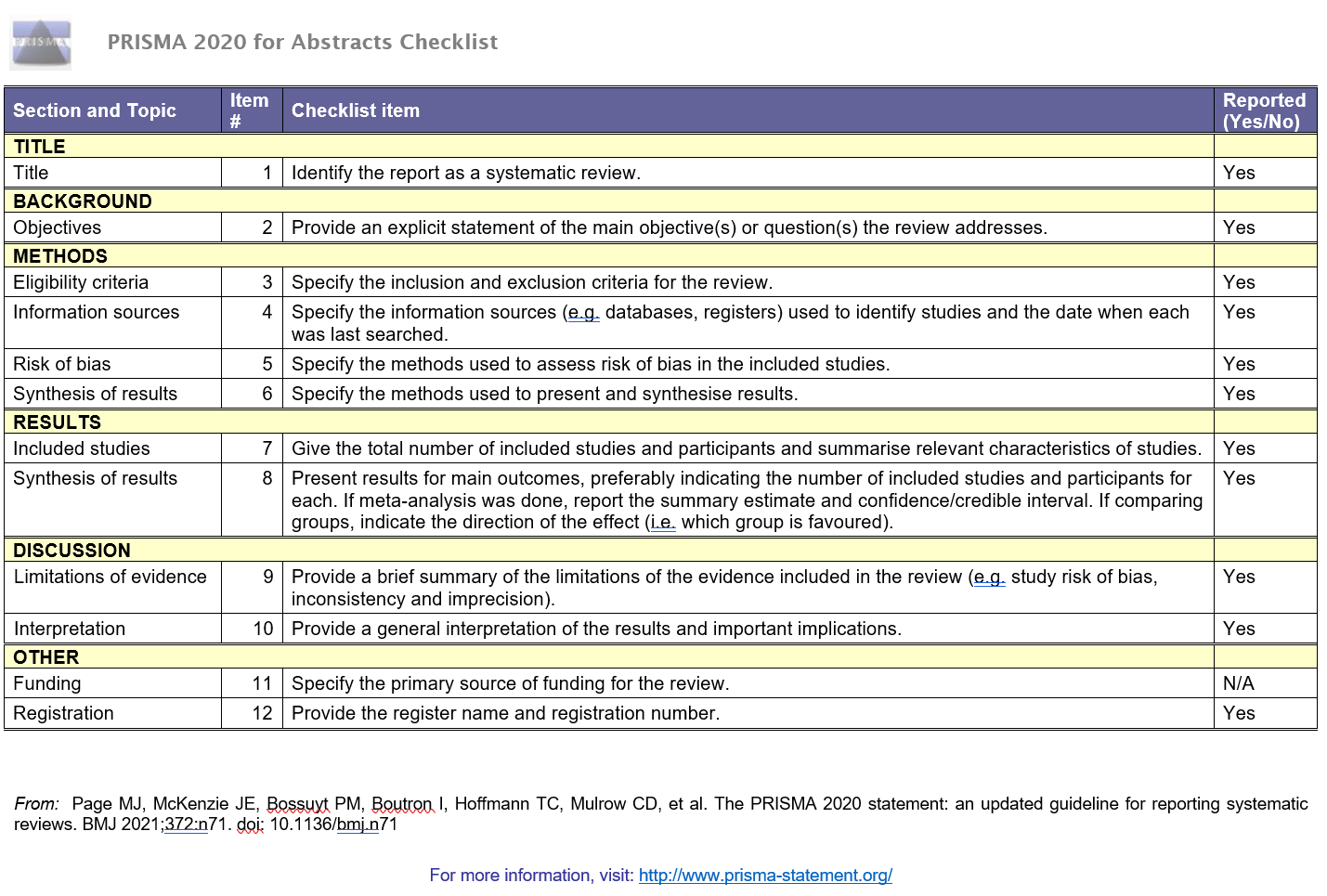


Appendix 2: The completed PRISMA checklists


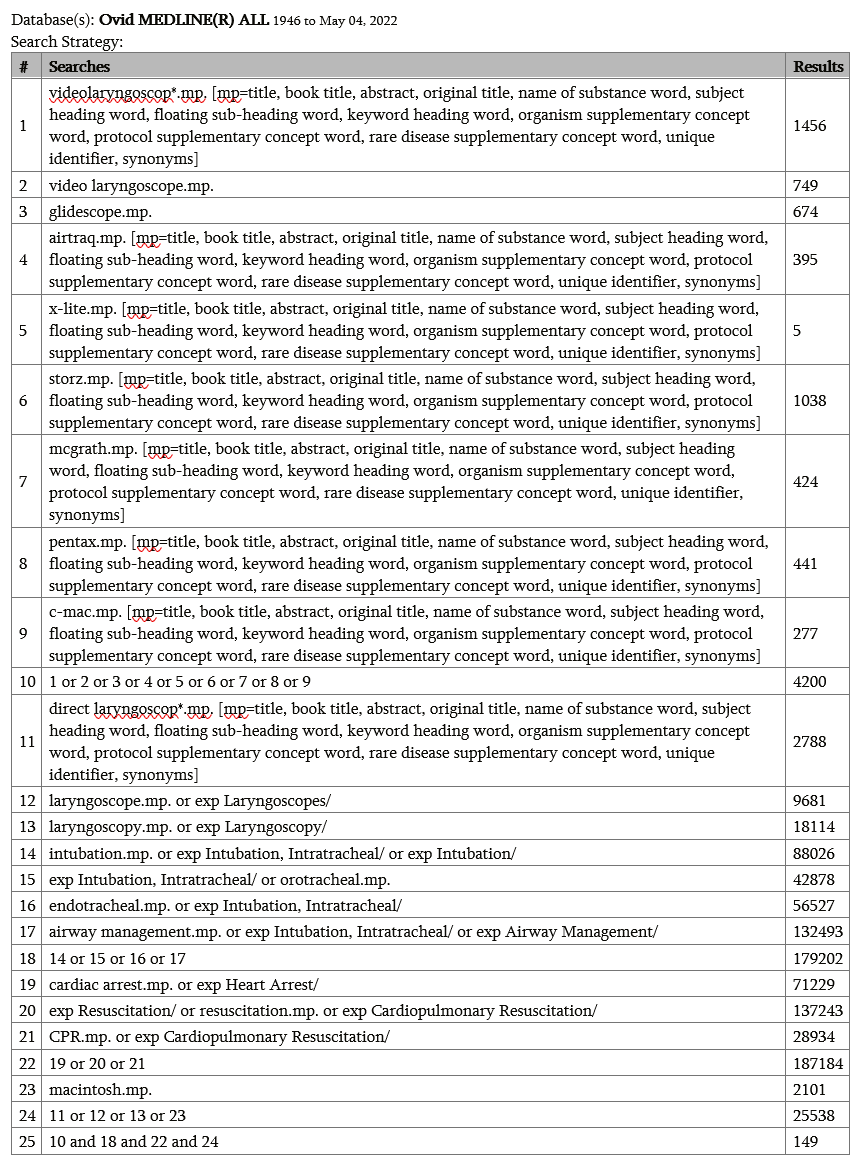


Appendix 3a: The search strategy as used for Medline


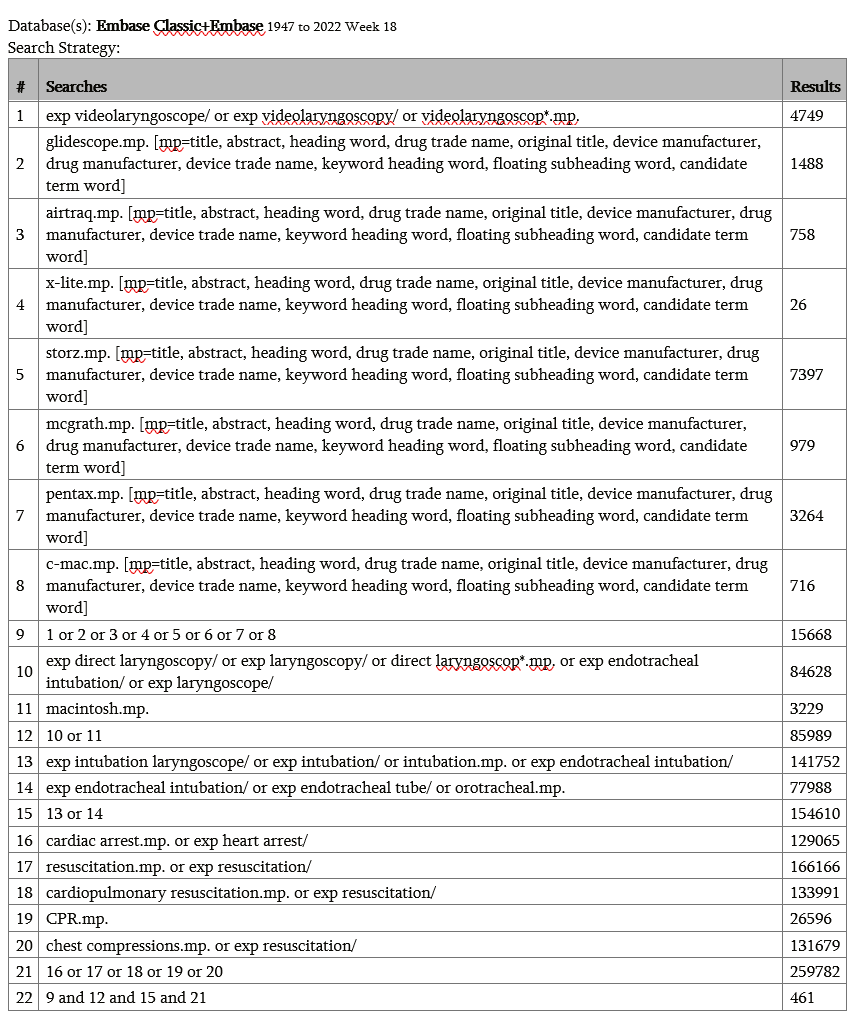


Appendix 3b: The search strategy as used for Embase


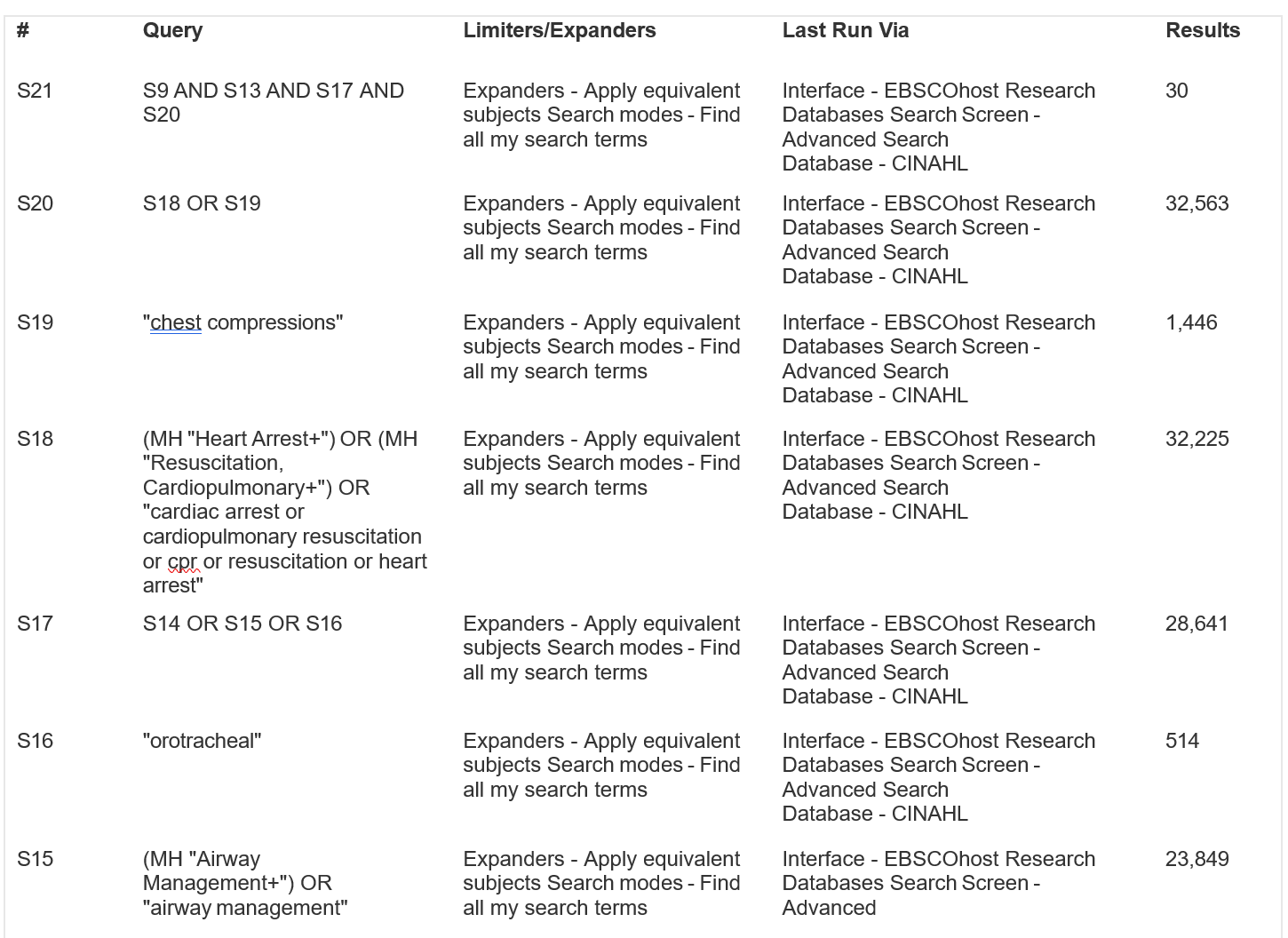


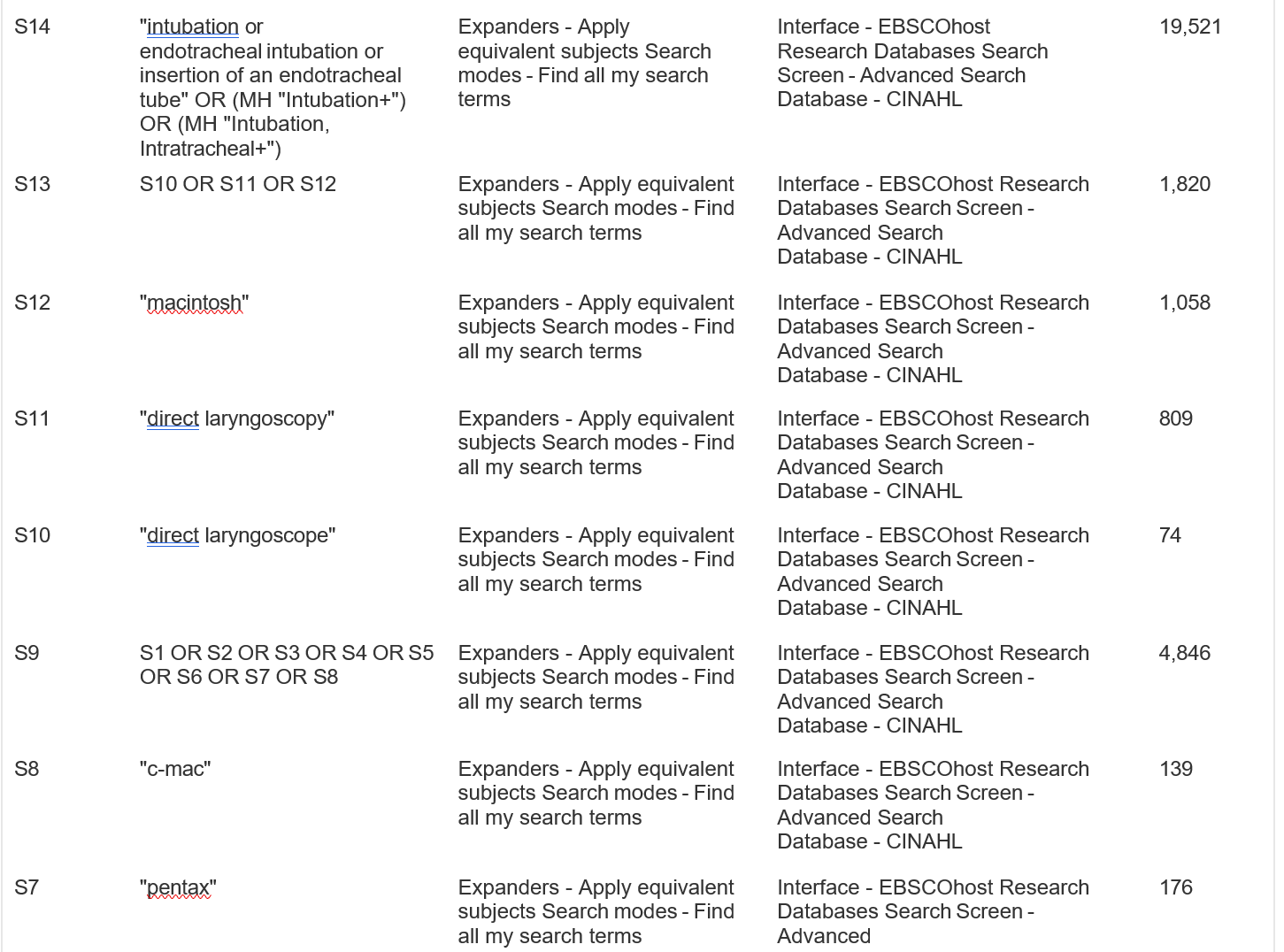


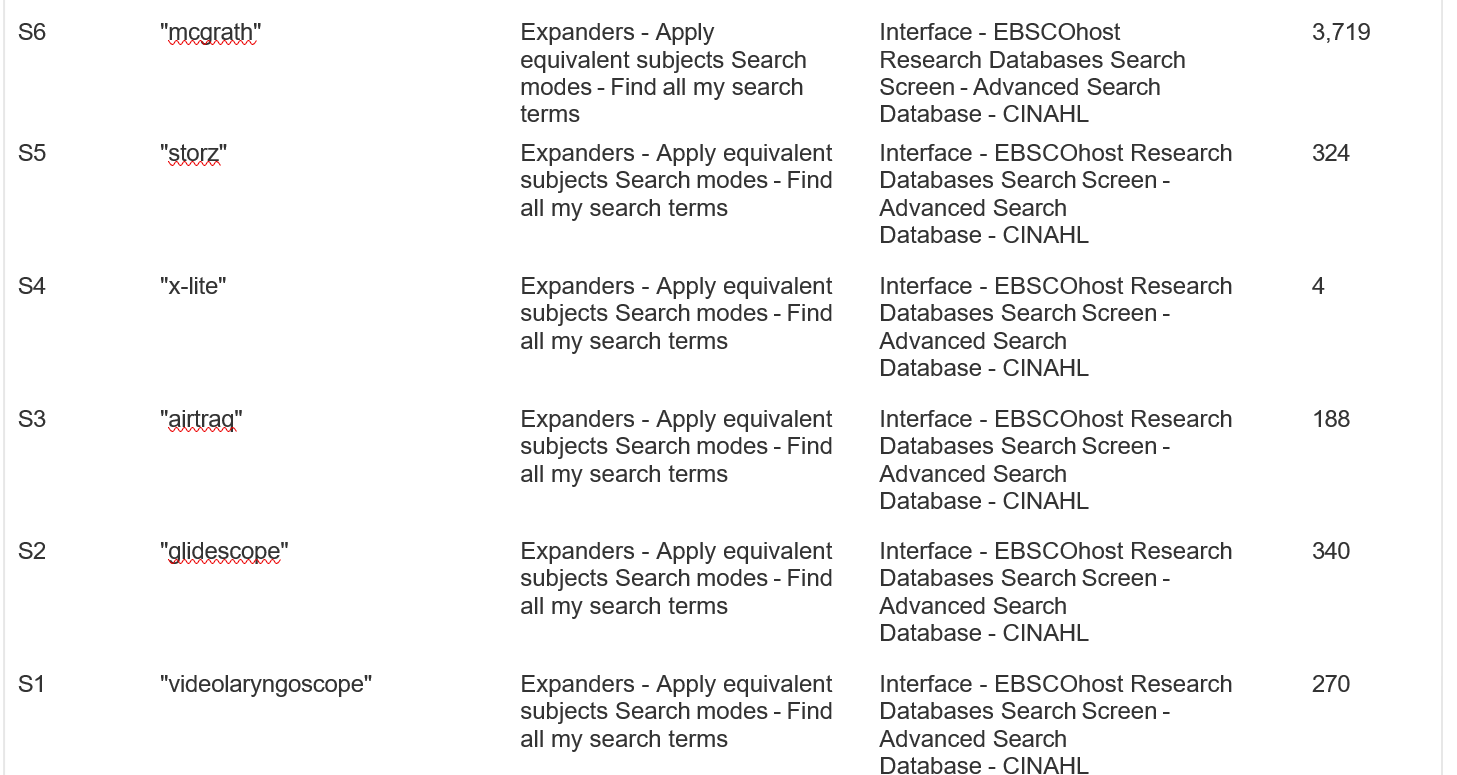


Appendix 3c: The search strategy as used for CINAHL


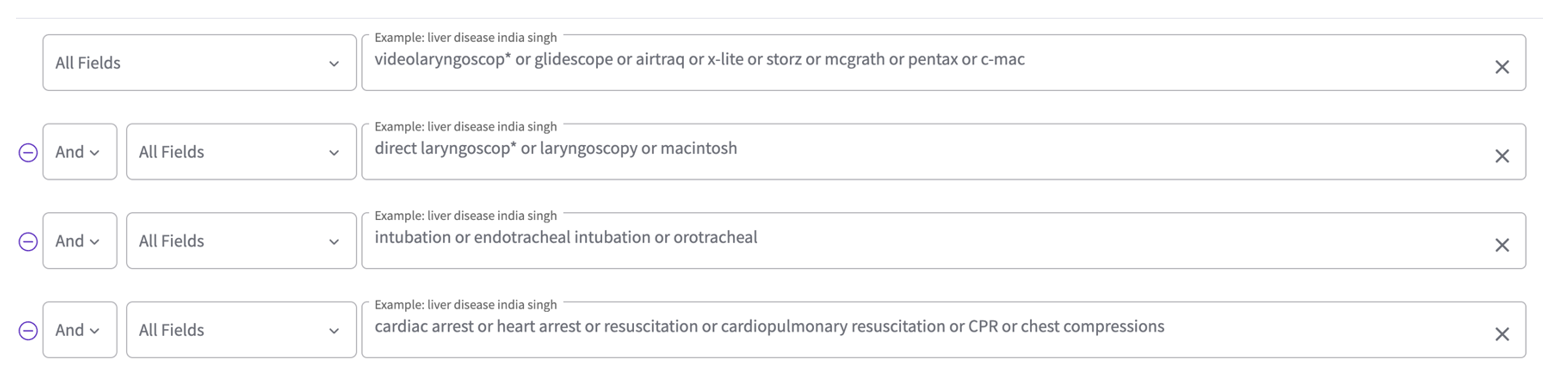


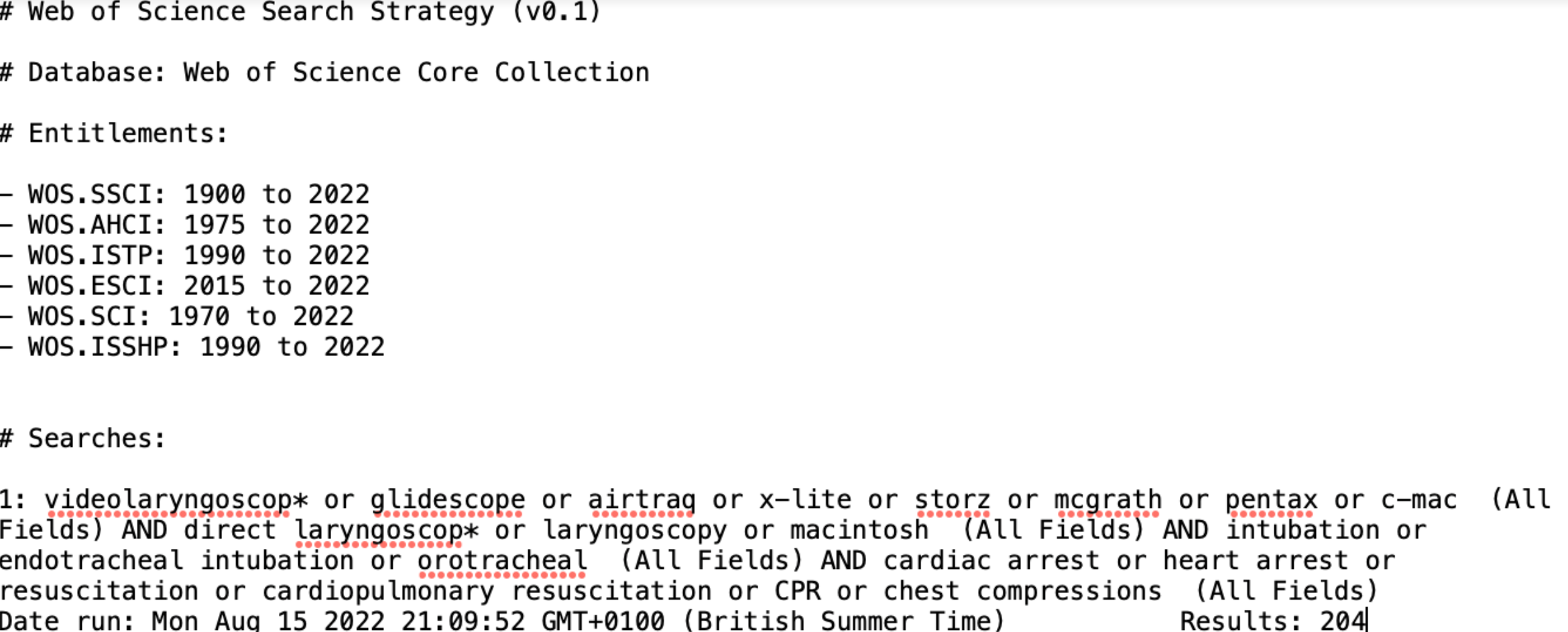


Appendix 3d: The search strategy and results summary (re-run in August 2022) as used for Web of Science

|  | Min et al. (2019) | Okamoto et al. (2019) | Lee et al. (2015) | Khandelwal et al. (2014) | Park et al. (2015) |
| --- | --- | --- | --- | --- | --- |
| Did the study address a clearly focused issue? | Yes | Yes | Yes | Yes | Yes |
| Was the cohort recruited in an acceptable way? | No – it wasn’t ideal recruitment, it was a retrospective study using data from a registry | Yes | No – it wasn’t ideal recruitment, it was a retrospective study using data from an electronic medical record | No – it wasn’t ideal recruitment, it was a retrospective study using data from an electronic medical record | Yes |
| Was the exposure accurately measured to minimise bias? | No – the choice of blade was at the discretion of the operator | No – the choice of blade was at the discretion of the supervising attending physician in the ED | No – the choice of blade was at the discretion of the operator. There were also 2 types of VL, but it wasn’t recorded which one was used | No – the choice of blade was at the discretion of the operator | Can’t tell – exposure was limited by year of the study, not all intubations were done by the residents so this could have resulted in selection bias |
| Was the outcome accurately measured to minimise bias? | No – the primary outcome of FPS rate was recorded by an independent observer and it wasn’t stated who this was or their level of expertise | No – an intubation attempt was defined as an insertion of the laryngoscope into the mouth, so looking inside the airway without attempting to pass a tube would have counted as a failed first attempt | Can’t tell – it wasn’t described what an attempt at intubation entailed | Can’t tell – it wasn’t described what an attempt at intubation entailed | Can’t tell – it wasn’t described what an attempt at intubation entailed |
| Have all important confounding factors been identified? | Yes | No – they didn’t account for skill of individual intubator | No – there was not accurate measurement of the experience of the operator | No – comorbidities weren’t accounted for | No – comorbidities weren’t accounted for, different operators were used in each group |
| Have the confounding factors been taken into account in the design/analysis? | Yes | Yes | Yes | Yes | Can’t tell – there was no mention of adjusting for variables during the analysis |
| Was the follow up of subjects complete enough? | Can’t tell | Can’t tell | Yes | Can’t tell | Can’t tell |
| Was the follow up long enough? | Can’t tell | Can’t tell | Yes | Can’t tell | Can’t tell |
| What are the results? | Higher FPS rate for VL when a junior resident | Higher FPS rate for VL if less experienced | Higher FPS rate for VL | No significant difference between VL and DL, most intubations were done by an experienced operator | Higher FPS rate for VL |
| How precise are the results? | Reported p-value 0.075  Confidence interval 1.17-2.77 | Reported p-value <0.001  Confidence interval 1.26-2.06  When adjusted for confounding factors, confidence interval 1.03-1.73 | Reported p-value 0.003  Confidence interval 1.30-4.50 | Reported p-value 0.27  Confidence interval 0.35-1.43 | Reported p-value <0.001  Confidence interval not reported |
| Do you believe the results? | Yes | Yes | Yes | Yes – the results agree with those from the other studies that FPS rate isn’t greatly impacted when the operator is experienced.  However, the proportion of patients intubated with VL was so low (5%) that it is hard to determine any significant effect | Yes – the results agree with those from other studies that FPS rate improves when the operator is a novice physician |
| Can the results be applied to the local population? | Can’t tell – they may be applicable to similar centres in that area, but unsure if the results can be generalised further due to operator differences in other countries | Can’t tell – comparisons between training and scope of practices would be needed for these results to be applied to the UK | No – it is not clear what the levels of operator experience were so it’s difficult to compare to the UK. It is unknown if there was a better VL between the 2. | No – the study setting could be applied to the UK, but the study design was not the most appropriate to answer the question | No – it was a very small study in a single hospital assessing less experienced physicians only, UK intubations are usually performed by more experienced physicians |
| Do the results of this study fit with other available evidence? | Yes | Yes | Can’t tell – other reports show a benefit of VL for inexperienced users, this report doesn’t differentiate between experience | Yes | Yes |
| What are the implications for practice? | VL shows higher FPS rates when used by junior residents, so this may be of use both in practice and for training purposes | VL may be of use when less experiences members of staff are intubating | VL may increase FPS rates, but it may not be able to be applied to practice due to the flawed data collection methods and lack of intervention randomisation | There was not sufficient evidence or sample size to deduct any important effects of VL vs DL | VL could have a place in training less experienced physicians, as they improved whilst using it over the year of the study |

Appendix 4a: Summary of the CASP analysis carried out for the full text papers using the cohort study checklist

|  | Kim et al. (2016) |
| --- | --- |
| Did the study address a clearly focused research question? | Yes |
| Was the assignment of participants to interventions randomised? | Yes |
| Were all participants accounted for? | Yes |
| Were participants/investigators/people assessing ‘blinded’? | Participants – yes  Investigators – no  People assessing – no |
| Were study groups similar? | Can’t tell – average ages were similar, there were more males in the DL group, other characteristics weren’t stated |
| Did each group receive the same level of care? | Can’t tell |
| Were the effects of the intervention reported comprehensively? | Yes |
| Was the precision of the intervention reported? | No – confidence intervals were not reported |
| Do the benefits of the intervention outweigh the harms and costs? | No – there was no significant difference reported in ETI success rates, number of attempts or total ETI time between DL and VL. There were however some harms noted in the control (DL) group – longer compression interruption and some serious no-flow events. Cost-effectiveness analysis was not undertaken, but it was noted that VL is expensive |
| Can the results be applied to your local population? | Yes |
| Would the experimental intervention provide greater value to the people in your care than any of the existing interventions? | Can’t tell – the only benefit shown is that of reducing compression interruption which should improve cardiac arrest outcomes, although these were not reported in this study |

Appendix 4b: Summary of the CASP analysis carried out for the full text paper using the randomised controlled trial checklist
